# Loss of prostatic acid phosphatase and α-synuclein cause motor circuit degeneration without altering cerebellar patterning

**DOI:** 10.1371/journal.pone.0222234

**Published:** 2019-09-11

**Authors:** Maryam Rahimi-Balaei, Matthew Buchok, Pirkko Vihko, Fiona E. Parkinson, Hassan Marzban

**Affiliations:** 1 Department of Human Anatomy and Cell Science, Max Rady College of Medicine, Rady Faculty of Health Sciences, University of Manitoba, Winnipeg, Manitoba, Canada; 2 The Children's Hospital Research Institute of Manitoba (CHRIM), Max Rady College of Medicine, Rady Faculty of Health Sciences, University of Manitoba, Winnipeg, Manitoba, Canada; 3 Department of Clinical Chemistry and Hematology, University of Helsinki, Helsinki, Finland; 4 Department of Pharmacology and Therapeutics, University of Manitoba, Winnipeg, Manitoba, Canada; University of Nottingham, UNITED KINGDOM

## Abstract

Prostatic acid phosphatase (PAP), which is secreted by prostate, increases in some diseases such as prostate cancer. PAP is also present in the central nervous system. In this study we reveal that α**-**synuclein (*Snca*) gene is co-deleted/mutated in *PAP* null mouse. It is indicated that mice deficient in transmembrane PAP display neurological alterations. By using immunohistochemistry, cerebellar cortical neurons and zone and stripes pattern were studied in *Pap*^*-/- ;*^*Snca*^*-/-*^ mouse cerebellum. We show that the *Pap*^*-/- ;*^*Snca*^*-/-*^ cerebellar cortex development appears to be normal. Compartmentation genes expression such as zebrin II, HSP25, and P75NTR show the zone and stripe phenotype characteristic of the normal cerebellum. These data indicate that although aggregation of PAP and SNCA causes severe neurodegenerative diseases, *PAP* ^*-/-*^ with absence of the *Snca* does not appear to interrupt the cerebellar architecture development and zone and stripe pattern formation. These findings question the physiological and pathological role of SNCA and PAP during cerebellar development or suggest existence of the possible compensatory mechanisms in the absence of these genes.

## Introduction

Alpha- synuclein (SCNA, 140 amino acids) is encoded by *Snca* gene and is present in the cytoplasm in both free and lipid associated forms [1,2]. This protein is one of several major members of intracellular fibrillary proteins, abundant protein in presynaptic axon terminals and important for brain normal function [3]. Synuclein family are comprised of α-, β-, and ɣ-synuclein, and synoretin [4]. It was found first in Torpedo californica’s acetylcholine vesicles and suggested to have a role in dopaminergic neurotransmission and synaptic plasticity [3,5]. When SNCA aggregates in the brain (forming oligomers and insoluble fibrils with increased ß-sheet configuration, Lewy bodies and non-Amyloid β component) it can result in a subset of neurodegenerative disorders like Alzheimer’s disease (AD), Parkinson’s disease (PD), dementia with Lewy bodies (DLB), and other synucleinopathies [6,7].

It is important to note that many of the proteins involved in the progression of neurodegeneration have crucial contributions during neurodevelopment [8]. In a study conducted on the developing human from fetus to adulthood, it is concluded that the expression of α-synuclein (SNCA) observed and condensed first in neuronal cell bodies of cortical plate at 11 weeks, then in the hippocampus, basal ganglia, and brain stem at 20 weeks and persist for few years after birth [9]. In the cerebellum, it expresses in granular layer and molecular layer which starts at 21 weeks and continues until adulthood [9]. In the study was done by Raghavan et al., it is demonstrated the expression of SNCA in brain (anatomical and subcellular localization) varies with age, and starts disappearing from the neuronal cytosol in early fetus, and only presents in neuronal processes in older fetuses and adults while its role changes from stem cell fate and differentiation to synapse plasticity, synaptogenesis, and neurotransmission [9].

In mice, the expression of SNCA in developing brain has been detected as early as E 9.5 in the marginal zone of the neocortex and later in the subplate [10]. In the cerebellum, the high expression of SNCA is firstly observed in the cerebellar nuclei and then, the same pattern is seen in the Purkinje cells. The reason for this spatio-temporal expression pattern underscored by the authors as a response to the neuronal migratory pathways and the formation of the synapse connections [10]. In the mice lacking SNCA, the regulatory role of SNCA is shown through depletion of the presynaptic vesicular pool [11].

Thiamine monophosphatase (TMPase) is known as a molecular marker of small-diameter dorsal root ganglia neurons [12]. It has been reported that transmembrane isoform of prostatic acid phosphatase (PAP) is identical to the TMPase [12]. The two types of PAP transcripts are generated by alternative splicing; transmembrane PAP (TMPAP) which consists of 11 exons (exon 1–9, 10a and 11), and cellular and secretory PAPs having 10 exons (exon 1–9 and 10b) [13–15]. TMPAP is a member of the plasma membrane-endosomal-lysosomal pathway and the length of the 3’ untranslated region of TMPAP is shorter than those of cellular and secretory PAPs: 405 bp versus 874 bp [15,16]. As an important marker for prostatic carcinoma, PAP was identified long before the introduction of prostate specific antigen (PSA) [14,17,18]. The expression of TMPAP was observed in nonprostatic tissues, including brain, kidney, liver, lung, skeletal muscle, placenta, salivary gland, spleen, thyroid, and thymus [19,20]. It is indicated that mice deficient in transmembrane PAP display increased GABAergic neurotransmission beside increase in striatal dopamine synthesis and neurological alterations [21]. Neurodegenerative disorders with synucleinopathies are accompanied with dopaminergic neuron loss [22]. Interestingly, PAP has stronger antinociceptive effects than morphine and has been suggested to use for the treatment of chronic pain [12].

In this study to question any neurological abnormalities related to cerebellum, we investigated the cerebellar cortex patterning to indicate any changes in form of stripes and patterns compartmentation in PAP mutant mice, to uncover the possible role of PAP and SNCA in the cytoarchitecture and function of the cerebellum.

## Material and methods

### Animal maintenance

All animal procedures were performed in accordance with institutional regulations and the *Guide to the Care and Use of Experimental Animals* from the Canadian Council for Animal Care and has been approved by local authorities “the Bannatyne Campus Animal Care Committee” (approved protocol # 15–066). *PAP* KO mice were obtained from Dr. Pirkko Vihko, University of Helsinki, Finland. All control wide types C57BL/6 mice were obtained from Central Animal Care Service, University of Manitoba. Animals were kept at optimum temperature and relative humidity (18–20°C, 50–60%) on a light and dark cycle (12:12 h) with free access to food and water. The midday of the vaginal plug discovery was designated embryonic day 0.5 (E0.5) and the day of birth postnatal day 0 (P0). Pregnant females (n = 6, 3 *Pap*^*-/-*^, 3 *Pap*^*+/+*^) were anesthetized with 40% isoflurane, UPS Baxter Co. Mississauga, Ontario, Canada) and killed via cervical dislocation. The embryos at E 12.5 were carefully dissected, placed immediately in ice-cold phosphate buffered saline (PBS) to remove blood, and then fixated overnight in the fixation solution (4% paraformaldehyde (PFA)). For postnatal brain sample collection, mice at P60 (n = 20; 10 *Pap*^*-/-*^, 10 *Pap*^*+/+*^) were anesthetized with isoflurane and transcardially perfused at first with ice-cold PBS and followed by the 4% PFA. Then brains removed from skull and placed in the same fixation solution for overnight.

### Sections immunohistochemistry and immunofluorescence

The embryos and postnatal brains were transferred to the gradient 10%-20%-30% sucrose until they sank at the bottom of the container. Then they were embedded in clear frozen section compound (OCT: VWR, USA), were frozen at -80°C and cut at 20 μm via cryostat microtome. The sections were placed on slides covered with a coating solution (0.05% chromic potassium sulfate and 0.5% gelatin) or floated in sterile PBS to be utilized for immunochemistry (IHC) process as explained in our previous studies [23,24]. Antibody dilutions were used as follows: SNCA (sc-69977, Santa Cruz) 1:500 [10], p75NTR (8238, Cell Signaling) 1:1000 [25]. Two anti-calbindin (Calb1) antibodies were used (in the cerebellum, Calb1 is entirely expressed in Purkinje cells): Rabbit polyclonal anti-calbindin D-28K antiserum (anti-Calb1, diluted 1:1,000, Swant Inc., Bellinzona, Switzerland), and mouse monoclonal anti-calbindin (anti-Calb1, diluted 1:1,000, Swant Inc., Bellinzona, Switzerland) [23,24]. Anti-zebrin II (a gift from Dr. Richard Hawkes, University of Calgary, Calgary, AB, Canada) is a mouse monoclonal antibody that was produced by immunization with a crude cerebellar homogenate from the weakly electric fish Apteronotus. We used it directly from spent hybridoma culture medium (diluted 1:200) [23,24,26]. Fluorescent detection was performed using followed antibodies: Alexa Fluor® 568 Goat Anti-Rabbit IgG (H+L), Alexa Fluor® 488 Chicken Anti Mouse IgG (H+L) (A-11036, A21200, Life Technologies) 1:1000. Detection of peroxidase IHC was also performed as described previously using HRP conjugated goat anti rabbit IgG and goat anti-mouse IgG (H+L) antibodies (EMD Millipore Corporation, 12–348 and AP308P, respectively) 1:500, and developed with DAB (3,3'-diaminobenzidine) solution (Sigma, St. Louis MO, USA).

### Western blotting analyses

Equal amount of proteins (n = 6, 3 *Pap*^*-/-*^, 3 *Pap*^*+/+*^) were separated by SDS/PAGE in 10–15% precast gels (Bio-Rad, Hercules, CA, USA) and transferred onto the PVDF-membrane. For the Western blot analysis, membranes were blocked in 5% nonfat dry milk (NFDM) in TBS containing 0.02% Tween 20 (TBST) and then incubated overnight at 4°C with primary antibodies as follows: α-synuclein (sc-69977, Santa Cruz) 1:2000. Secondary antibodies as follows: HRP conjugated goat anti-mouse IgG (AP308P, Millipore) 1:6000. Binding was assessed using DAB (3,3'-diaminobenzidine) solution (Sigma, St. Louis MO, USA).

### PCR analysis

To study the expression of SNCA in *Pap*^*-/-*^and *Pap*^*+/+*^, total DNA from cerebellum (n = 6, *Pap*^*-/-*^, 3 *Pap*^*+/+*^) was extracted using a kit (AccuStart^TM^ II Mouse Genotyping Kit, Cat# 95135–500, Quanta BioSciences, Inc. MD, USA), according to manufacturer’s instructions. DNA quality and quantity were determined by measuring the absorbance at 260 and 280 nm using NanoDrop ND-1000 UV- 19 Vis Spectrophotometer (Thermo Fisher Scientific, Waltham, MA, USA). All samples had an absorption ratio A260/A280 between 1.8 and 2.2. DNA (1 μg) from each sample was used.

To amplify the SNCA gene, PCR reactions were performed in a T3000 thermocycler (Biometra, Göttingen, Germany) using AccuStart^TM^ II GelTrack^TM^ PCR SuperMIX (2X) (Cat# 95136–500, Quanta BioSciences, Inc. MD, USA) in a final volume of 25 μL. There were three sets of primers to amplify across an intron to probe genomic DNA for SNCA (the SNCA primer sequences are presented in S2 Fig). Then PCR products were run on PCR agar gel and detected for the target gene (SNCA) bands to distinguish differences in PAP-WT from PAP-KO mice.

### Imaging and figure preparation

For bright field microscopy, images were captured using Zeiss Axio Imager M2 microscope (Zeiss, Toronto, ON, Canada). Images were then analyzed with a Zeiss Microscope Software (Zen Image Analyses software) (Zeiss, Toronto, ON, Canada). For fluorescence microscopy of the embryonic entire cerebellum sections, a Z2 Imager Zeiss Fluorescence microscope (Zeiss, Toronto, ON, Canada) equipped with a camera was used to capture the images. Images were then analyzed using Zen software. Images were cropped, corrected for brightness and contrast, and assembled into montages using Adobe Photoshop CS5 Version 12.

## Results

### SNCA expression in cerebellum of *PAP*^*-/-*^ mouse and wide type controls (Zone and Stripe Pattern)

**Fig 1:**
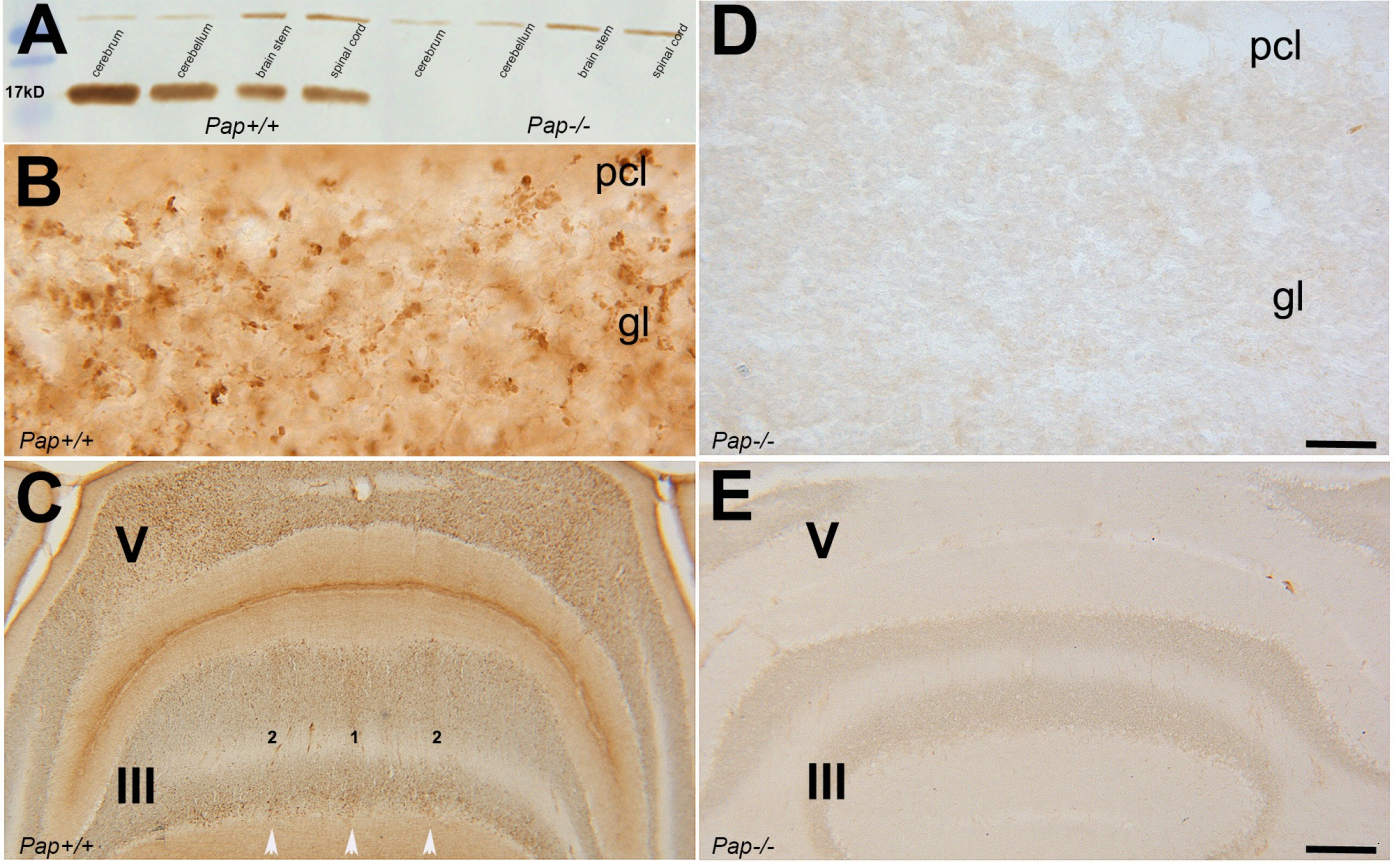
SNCA expression in cerebellar vermis of adult WT and *Pap* null mice. **A**. Immunoblotting showed lack of the SNCA expression in *Pap* null cerebellum, cerebrum, brainstem, and spinal cord tissue at P = 60 in comparison to the control. **B-C.** SNCA immunoperoxidase staining of a transverse section through the anterior cerebellum at P = 60 shows strong immunoreactivity in the granular layer in the terminals of mossy fibers afferents (B) in an array of parasagittal stripes in anterior zone (C). **D-E.** The *Pap* null cerebellum shows a lack of SNCA expression at low (D) and in higher magnification (E). Abbreviations: pcl: Purkinje cell layer, gl: granular layer, III and V: lobule III and lobule V Scale bar: 20 μm in B and D; 200 μm in C and E.

The expression of SNCA in different part of central nervous system (cerebrum, cerebellum, brain stem and spinal cord) was evaluated at protein level by Western blotting. Western blot analysis at P = 60 showed SNCA expression in all different parts (cerebrum, cerebellum, brain stem and spinal cord) of the control *Pap*^*+/+*^ and none of the *PAP*^*-/-*^ (Fig 1A). The expression of SNCA was also evaluated by immunohistochemistry of transverse cerebellar sections at P = 60 and confirmed Western blot results. In the cerebellum of control *Pap*^*+/+*^, SNCA was expressed in the axon terminals of the mossy fibers in the granular layer (Fig 1B). Transverse section through the anterior cerebellum immunoperoxidase-stained for SNCA showed strong immunoreactivity in the granular layer in an array of parasagittal stripes (lobules III and V; Fig 1C). The *PAP*^*-/-*^ transverse cerebellar section was immunolabeled with SNCA and showed no immunoreactivity in granular layer (Fig 1D) or in anterior zone (Fig 1E).

### The expression of SNCA in embryonic stage in *PAP*^*-/-*^ mouse and wide type controls

**Fig 2:**
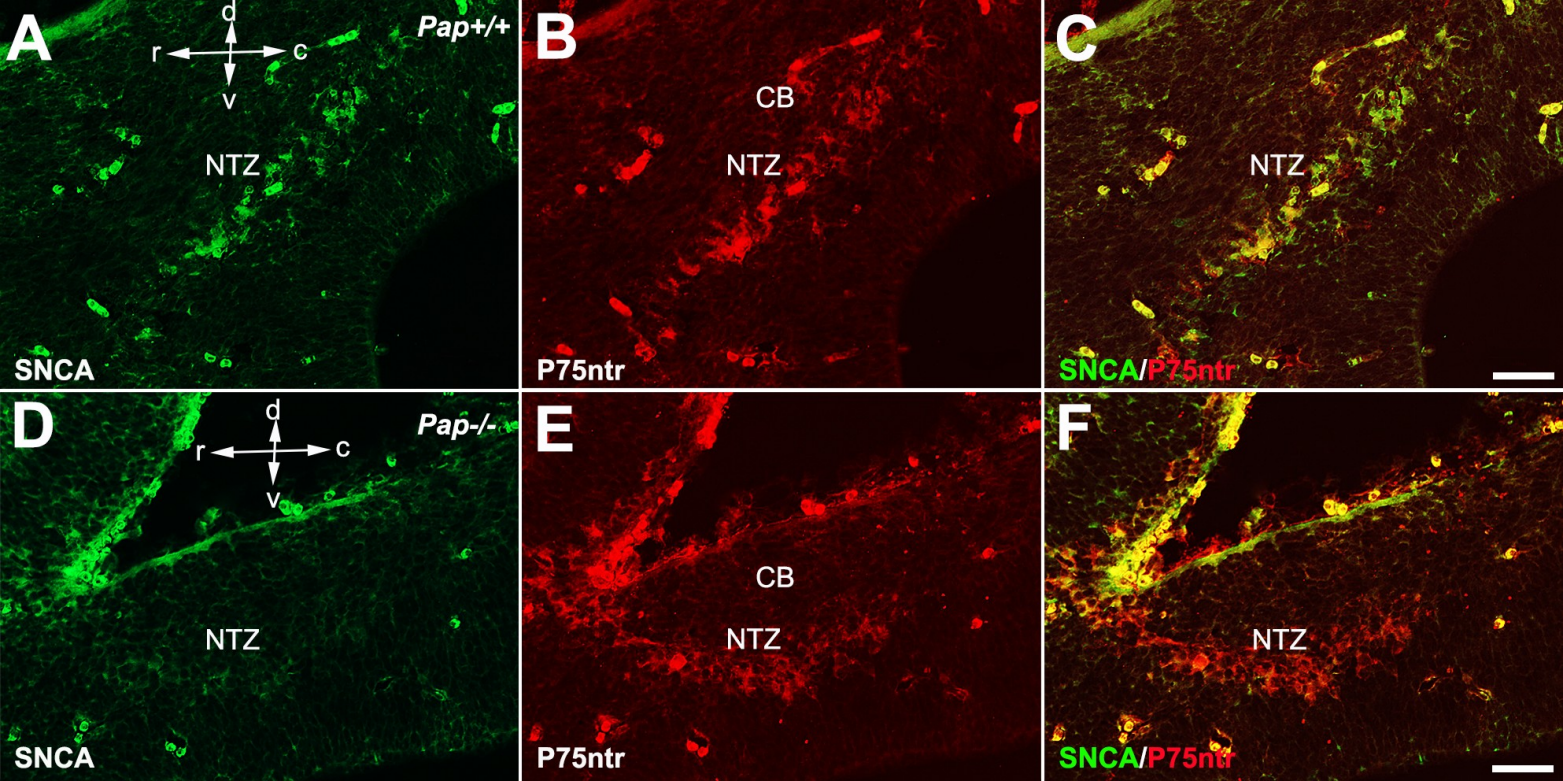
Lack of *Snca* expression in the *Pap* mouse at E12. **A-F**. Double immunostaining with SNCA (green) and P75NTR (red) at E12 in WT (A–C) and *Pap*-null mice (D–F) shows a lack of SNCA expression in NTZ cells (D), while P75NTR cells are present in NTZ cells and confirm the presence of these cells (E). Abbreviations: CB: cerebellum, NTZ: nuclear transitory zone Scale bar = 50 μm.

To determine the expression of SNCA during early development, double immunofluorescence staining performed in sagittal cerebellar sections at E12 with SNCA and P75NTR (positive immunoreactivity for nuclear transitory zone (NTZ) at early cerebellar development). In control *Pap*^*+/+*^ (Fig 2A–2C), SNCA immunopositive cells in NTZ (Fig 2A) and P75NTR immunopositive cells in NTZ (Fig 2B) were observed and co-labeled (merge one, Fig 2C). In comparison, the *PAP*^*-/-*^ sagittal cerebellar sections at E12 showed no immunoreactivity for SNCA (Fig 2D) while P75NTR immunopositive cells were seen in NTZ (Fig 2G). No co-labeling of NTZ was seen (Fig 2F).

### PCR results

**Fig 3:**
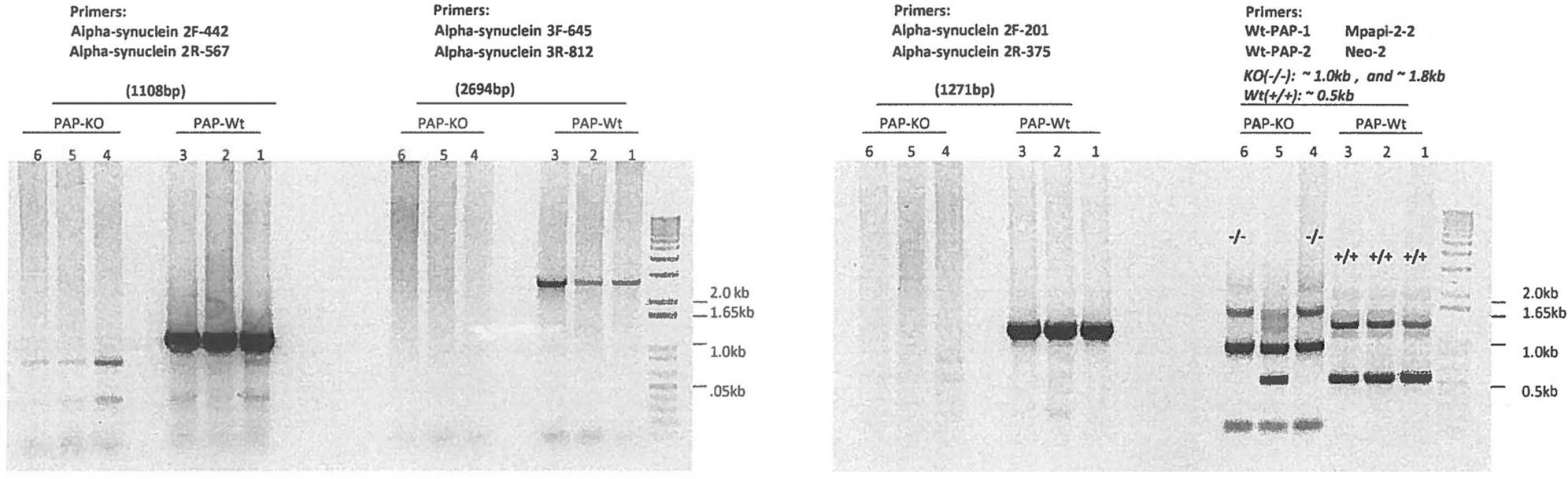
The three sets of primers, each to amplify across an intron to probe genomic DNA for *Snca*, which are all were negative in the *Pap KO* mice and positive in the WT mice. PCR amplification of α -synuclein from *Pap KO* and WT mouse genomic DNA to verify that the gene is present. From intron-exon map, it is feasible to amplify from exon 1–281 to exon 281–430 (intron is 1096); exon 426–470 to exon 469–614 (intron is 2586); exon 612–698 to exon 698–1286 (intron is 940). Primer pair 1- PCR product size is 1271, Primer pair 2- PPCR product size is 2694, and Primer pair 3- PCR product size is 1108. The data convincing show that the expected PCR products are seen in WT but not *Pap KO* genomic DNA.

To confirm the lack of *Snca* in *Pap*^*-/-*^, the expression of *Snca* in *Pap*^*-/-*^ and *Pap*^*+/+*^ was studied by amplification across an intron to probe genomic DNA for *Snca*. We found that *Snca* was deleted in *Pap*^*-/-*^ mice after running PCR products on agar gel with three sets of different primers of SNCA (Fig 3 and S1 Fig).

### The expression of Calb1 and zebrin II in cerebellum of *Pap*^*-/-*^ *; Snca*^*-/-*^ mouse and wide type controls (Zone and Stripe Pattern)

**Fig 4:**
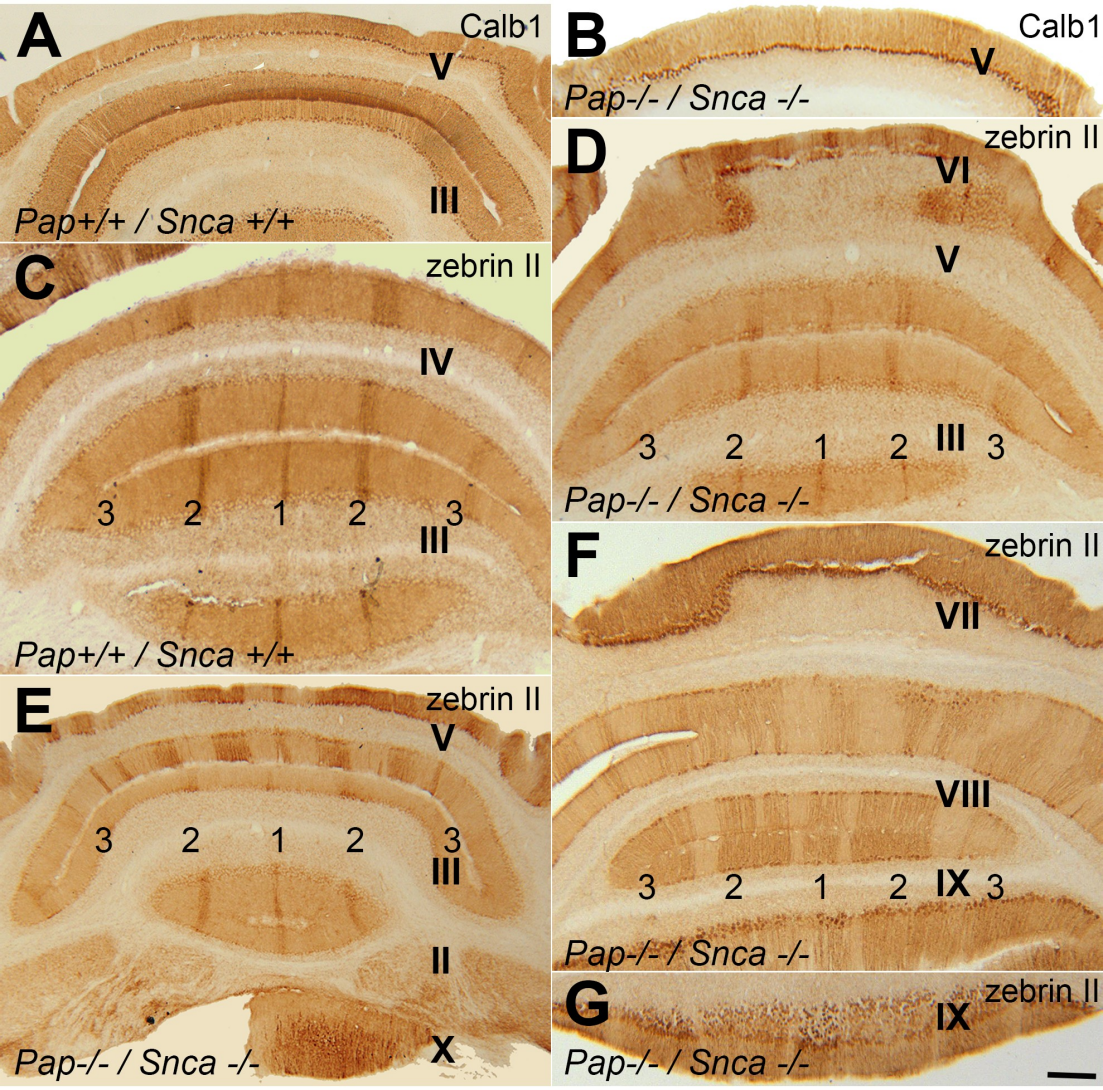
Transverse sections through the adult cerebellum of WT and *Pap* null mice immunostained with Calb1 and zebrin II. **A-B.** Immunohistochemistry with Calb1 (a pan Purkinje cell marker in the cerebellum) shows there is no phenotypic alteration between the WT (A) and *Pap* mutant (B). **C-G.** Transverse sections through the cerebellum of the adult WT (C) and *Pap* null (D-G) immunoperoxidase stained for zebrin II. The pattern of parasagittal stripes in the anterior zone central zone, posterior zone, and nodular zone are normal. The conventional stripe nomenclature has zebrin II+ stripes called P+, and are numbered from P1+ (indicated by 1) at the midline to P3+ (indicated by 3) laterally(e.g., [30] and [65]). Abbreviations: Scale bar = 200μm.

The expression of Calb1 (a marker expressed by all of the Purkinje cells [23]) and is a critical determinant of the precision of motor coordination [27] was also evaluated by immunohistochemistry of transverse cerebellar sections at P = 60 and showed no differences between *Pap*^*+/+*^ *; Snca*^*+/+*^ (Fig 4A) and *Pap*^*-/-*^ *; Snca*^*-/-*^ (Fig 4B) and appeared all Purkinje cells are present with normal phenotype, arranged Purkinje cell bodies in line and their dendrites were arborized to molecular layer. Immunolabeling of transverse cerebellar sections with zebrin II showed that the pattern expression of zebrin II with parasagittal stripes in *Pap*^*+/+*^ *; Snca*^*+/+*^ (Fig 4C) is the same in anterior (Fig 4D), central (Fig 4E), posterior (Fig 4F) and nodular (Fig 4G) zones in *Pap*^*-/ -*^*; Snca*^*-/-*^ mice and is normal. The mouse cerebellar cortex is subdivided into four transverse zones—the anterior zone (AZ: ~lobules I–V), the central zone (CZ: ~lobules VI–VII, with two sub-zones—see [28]), the posterior zone (PZ: ~lobules VIII + dorsal IX), and the nodular zone (NZ: ~ventral lobule IX + lobule X) (e.g., [29] and [30]).

### The expression of P75NTR and HSP25 in cerebellum of *Pap*^*-/ -*^*; Snca*^*-/-*^ mouse and wide type controls (Zone and Stripe Pattern)

**Fig 5:**
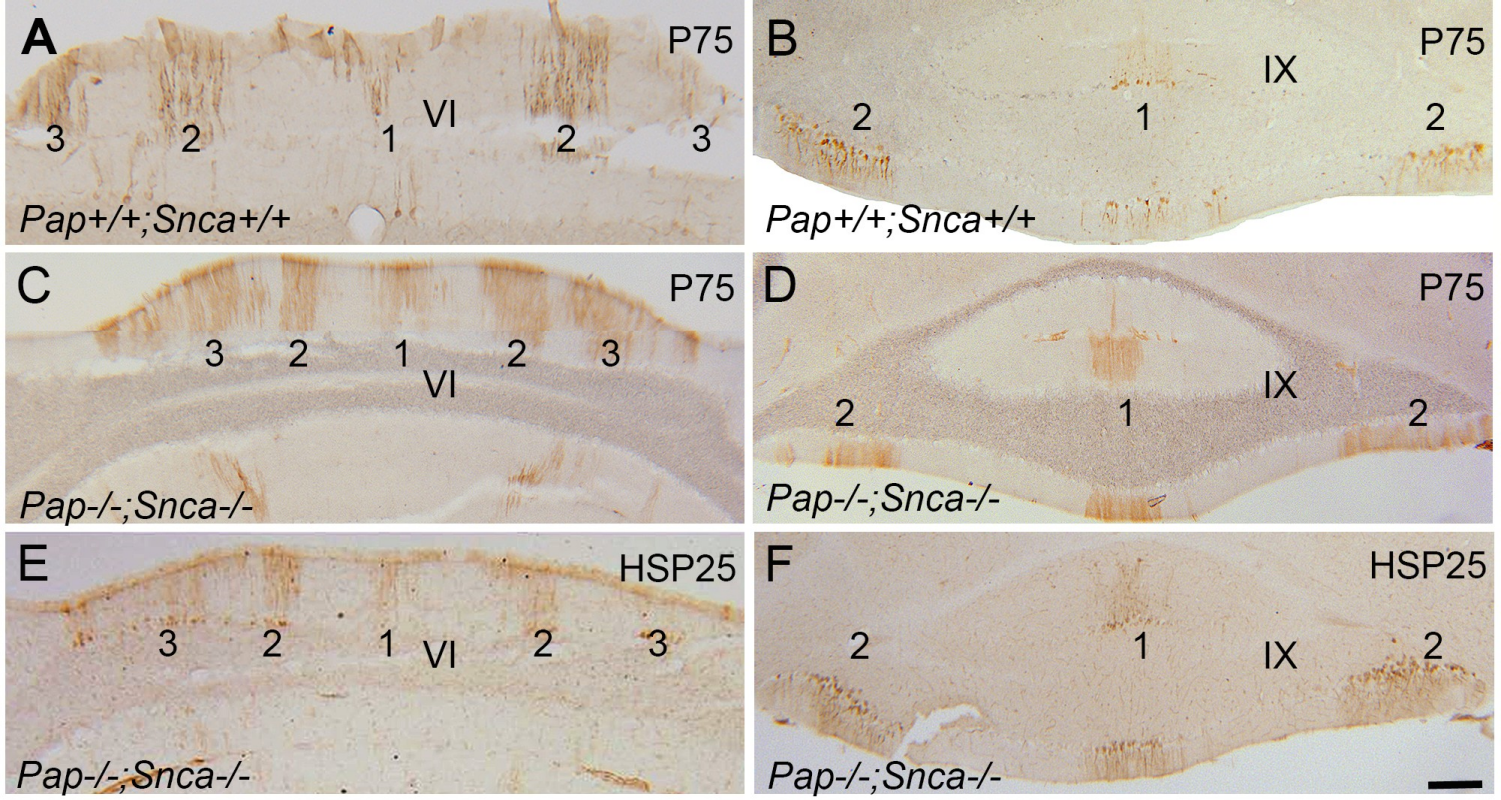
**P75NTR and HSP25 expression in the cerebellum of the WT (A-B) and *Pap* null mouse (C-F). A-B.** P75NTR immunoperoxidase staining shows pattern of stripe immunoreactivity in central zone (A) and nodular zone (B) in WT cerebellum. **C-D.** P75NTR immunoperoxidase staining shows pattern of stripe immunoreactivity in central zone (C) and nodular zone (D) in *Pap* null cerebellum which is comparable with WT. **E-F.** Immunoperoxidase staining for HSP25 in transverse sections through the central zone and nodular zone of the *Pap* mutant cerebellum reveals that subsets of Purkinje cells expressing HSP25 are interposed by HSP25 immunonegative Purkinje cells which are comparable with p75NTR expression in WT and *Pap* mutant. Abbreviations: Scale bar = 200μm.

The expression of P75NTR in central zone (Fig 5A) and nodular zone (Fig 5B) in *Pap*^*+/+*^ *; Snca*^*+/+*^ transverse sections of cerebellum at P = 60 showed immunoreactivity in stripes pattern. In comparison to the *Pap*^*+/+*^ *; Snca*^*+/+*^, the transverse sections of *Pap*^*-/ -*^*; Snca*^*-/-*^ cerebellum showed similar pattern of stripes with P75NTR immunopositive Purkinje cells in central (Fig 5C) and nodular zone (Fig 5D). The pattern of P75NTR positive immunoreactivity resembles HSP25 expression in same stripes pattern in central and nodular zone of *Pap*^*-/—*^*; Snca*^*-/-*^ cerebellum (Fig 5E and 5F). HSP25 immunolabeling is absent in the anterior and posterior zones, while is expressed in parasagittal stripes in the central and nodular zone of the normal cerebellum [23].

## Discussion

In this study, we have shown that despite the importance of the expression of PAP and SNCA during development, knock-out mice have normal brain morphology, including stripes and zone patterns of the cerebellar cortex architecture. An initial objective of the project was to identify the expression of SNCA in *PAP* mouse, and the idea of studying the expression of SNCA in *Pap KO* mice was done following our lab interest in cerebellar expression of SNCA during early and postnatal development. One interesting finding is that at different part of the brain (cerebrum, cerebellum, brain stem and spinal cord) there was no expression of SNCA in the *Pap* mouse. SNCA is not expressed in the axon terminals of mossy fibers and confirms the absence of this gene in *Pap* mouse. From our data, it appears that *Pap KO* is also *Snca* knock out. It is hard to call them double knock out since it was not done intentionally. Several reports have shown their findings on the deletion of *Snca*. In studies done on the mutated model of *Snca*, it is indicated that although *Snca* is expressed in all brain regions, knock-out mice have normal brain morphology and cellular structure. However, these mice demonstrates Lewy bodies inside the dopaminergic neurons under certain conditions. It is important to note that abnormal dopamine signaling may be associated with the role of SNCA in the nigrostriatal pathway, which is functional and not structural [31,32]. Further, other studies have shown that decreased dopamine levels in the striatum and reduced locomotor activity in response to amphetamine plus resistance to toxic effects of MPTP (a neurotoxin used to induce PD in animals by destroying dopaminergic neurons) [33–35].

As indicated before by Zhong et al. during embryonic stage, SNCA is condensed in NTZ of early developing cerebellum [10]. To examine the possibility of the disappearance of NTZ cells in the current study we found that despite the deletion of this gene, NTZ cells are still there and are labeled by P75NTR, while are immunonegative for SNCA. Neuronal proliferation/differentiation is regulated by P75NTR that are mainly expressed during early development [36–38].

Another interesting finding was by PCR experiment and confirmed the simultaneous deletion of *Snca* with *Pap* while a note of caution is due here since questioning the possibility of deletion of other genes and mainly raises questions about the original strain and / or mutation method.

There is a fundamental and unique cytoarchitecture organization in the cerebellar compartmentation and each gene is expressed, functioned and aligned in the pattern of zones and stripes [23–25,39–41]. The best way of studying Purkinje cell degeneration and vulnerability is indicated by the pattern of gene expression in different cerebellar lobules [23,25,39,40,42,43]. Many genes are expressed uniformly or their immunoreactivity are negative in the CZ and NZ [23,44]. Here, well-known Purkinje cell markers were used: Calb1, zebrin II, P75NTR and HSP25. The expression pattern of Calb1 showed no differences between normal and mutant mice and was expressed in all of Purkinje cells. The parasagittal striped expression pattern of zebrin II with immunopositive bands from medial to lateral of adult transverse cerebellar sections are termed P1+ to P7+ [25,45] and were identical in different zones. HSP25 is another Purkinje cell marker and it is expressed in the CZ and NZ uniformly at around P12 [46]. By around P15-21 the expression pattern of HSP25 in CZ became striped. It is reported the corticogenesis and development of CZ occurred in slower pace in comparison to the other cerebellar zones [47]. In our previous study, we have shown that the pattern of P75NTR expression in the adult cerebellar tissue is comparable with HSP25 expression [25]. Surprisingly, the parasagittal stripe pattern expression of P75NTR and HSP25 in the CZ and NZ resembled each other and showed no differences between knockout and WT control cerebellum. All in all were in support of the normal cerebellar compartmentation and morphology in dual *Pap / Snca KO* mice.

Small-diameter neurons are located in the dorsal root ganglia and trigeminal ganglia which are responsible for sensing painful and tissue-damaging stimuli [48,49]. These neurons express acid phosphatase or TMPase [50,51]. Prostatic acid phosphate is used as an indicator for diagnosis and treatment response in prostate carcinoma patients [14,18,52,53]. It has been shown that there is a decrease in thiamine-dependent processes in the postpartum brains of patients with neurodegenerative diseases. Evidence indicates that antinociceptive effects of thiamine are mediated by PAP [54]. Decrease in thiamine-dependent enzymes (TMPase, transmembrane isoform of PAP [15]) result in the antioxidant reversal and increased oxidative and nitrosative stress lead to neurodegenerative disease [55]. In addition, thiamine-dependent processes play an important role in glucose metabolism. Interestingly, AD and thiamine (vitamin B1) deficiency are associated with reduced glucose metabolism and increased oxidative stress in the brain. These two disorders are accompanied by irreversible cognitive impairment and share similar behavioral consequences, but are not identical and implicate the involvement of thiamine in the modulation of peroxisomal function, oxidative stress, protein processing, and transcription [56,57]. Beside role of PAP in neurodegeneration, it is reported that *Pap* knockout mice have normal acute pain sensitivity but enhanced sensitivity in chronic inflammatory and neuropathic pain models (increased thermal hyperalgesia and mechanical allodynia) [12]. In addition, PAP introduced as a neglected ectonucleotidase (extracellular adenosine production by dephosphorylation of the extracellular AMP to adenosine) could regulate diverse physiological processes that are dependent on adenosine [58,59]. Both adenosine and oxidative stress play an important role in prostate cancer [60,61]. TMPase deficient mice display increased GABAergic transmission and neurological alterations [21].

The most interesting aim of this experiment to pursue was the lack of the alpha-synuclein protein (SNCA) which is perhaps a consequence of the inbreeding in *Pap KO* mice. Specht and Schoepfer has shown that the deletion of alpha-synuclein locus is shown in the C57BL/6S strain mice, namely animals from Harlan when they inbred [62]. Interestingly, It is indicated that after *Pap KO* mice was generated, the homogenous background obtained by backcrossing to the C57BL/6 strain (Harlan Laboratories Inc.) for 16 generation [63,64].

These findings demonstrate the normal appearance of the stripe and zone patterns of the genes involved in cerebellar cortex architecture and compartmentation in *Pap*^*-/ -*^*; Snca* mice and if there is an unintentional double knockout, it has been raised an important issue for future research; what else is missing?

## Conclusion

To conclude, the most obvious finding to emerge from this study is that *Pap* and *Snca* dual KO mice have normal cerebellar morphology. These findings will be of interest to clinicians who are working on Parkinson’s disease, Alzheimer’s disease or other synucleinopathies.

## Supporting information

S1 Fig The DNA expression of *Snca* and *Pap* in control and KO mice embryos. PCR amplification of *Snca* and *Pap* in *KO* and WT mouse embryos genomic DNA to verify that the gene is present or not. *Snca* primer pair 3- PCR product size is 1108. The data convincing show that the expected PCR products are seen in WT but not *Pap KO* genomic DNA. *Pap* primer pair PCR product size is ~ 1000 (oligonucleotides used, (5´-TGCTGCACGGATACACATGC-3´ and 5´-TCGCAGCGCATCGCCTTCT-3’)) and *WT* primer pair PCR product size is ~ 500 oligonucleotides used (5´- GCA TGG AAC AGC ACT ACG AAC T -3´ and 5´- TCC ACA TCT GTG CTC CGG ATA T -3’). The data show that the expected PCR products are seen in *WT* at around 500 in WT mice and for *Pap* at around 1000 in *KO* genomic DNA.
(TIF)

S2 Fig *Snca* primers for cDNA and genomic DNA. In this study, the primers sequences were designed to be useful for both cDNA and genomic DNA, with small products using cDNA and bigger products using genomic DNA.
(TIF)
