# Supplementary figures and images for: Loss of prostatic acid phosphatase and α-synuclein cause motor circuit degeneration without altering cerebellar patterning
Source: PLoS One. 2019 Sep 11;14(9):e0222234. doi: 10.1371/journal.pone.0222234 (PMC6738605; doi:10.1371/journal.pone.0222234)

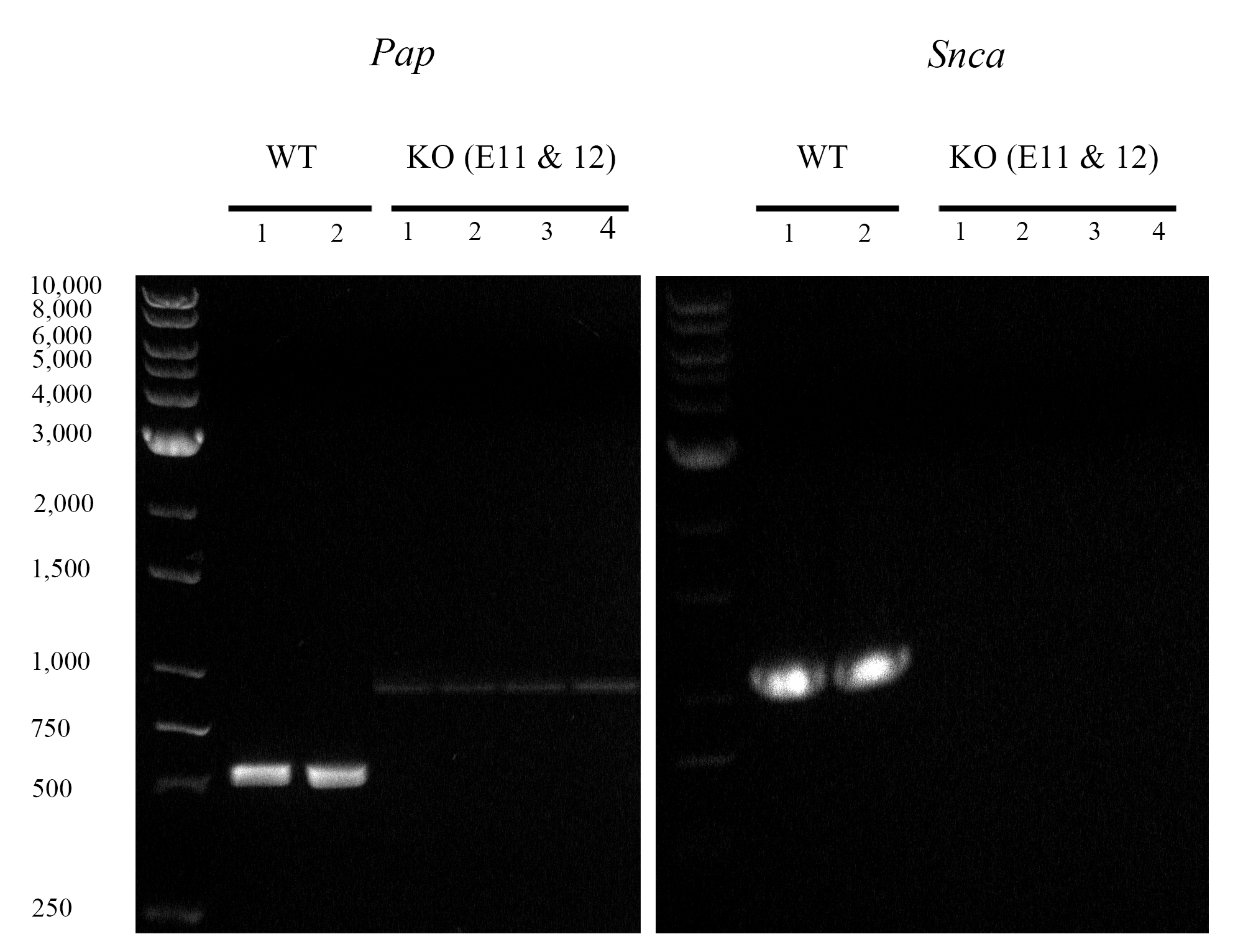

Supplement: S1 Fig — PCR amplification of Snca and Pap in KO and WT mouse embryos genomic DNA to verify that the gene is present or not. Snca primer pair 3- PCR product size is 1108. The data convincing show that the expected PCR products are seen in WT but not Pap KO genomic DNA. Pap primer pair PCR product size is ~ 1000 (oligonucleotides used, (5´-TGCTGCACGGATACACATGC-3´ and 5´-TCGCAGCGCATCGCCTTCT-3’)) and WT primer pair PCR product size is ~ 500 oligonucleotides used (5´- GCA TGG AAC AGC ACT ACG AAC T -3´ and 5´- TCC ACA TCT GTG CTC CGG ATA T -3’). The data show that the expected PCR products are seen in WT at around 500 in WT mice and for Pap at around 1000 in KO genomic DNA. (TIF) [file pone.0222234.s001.tif]

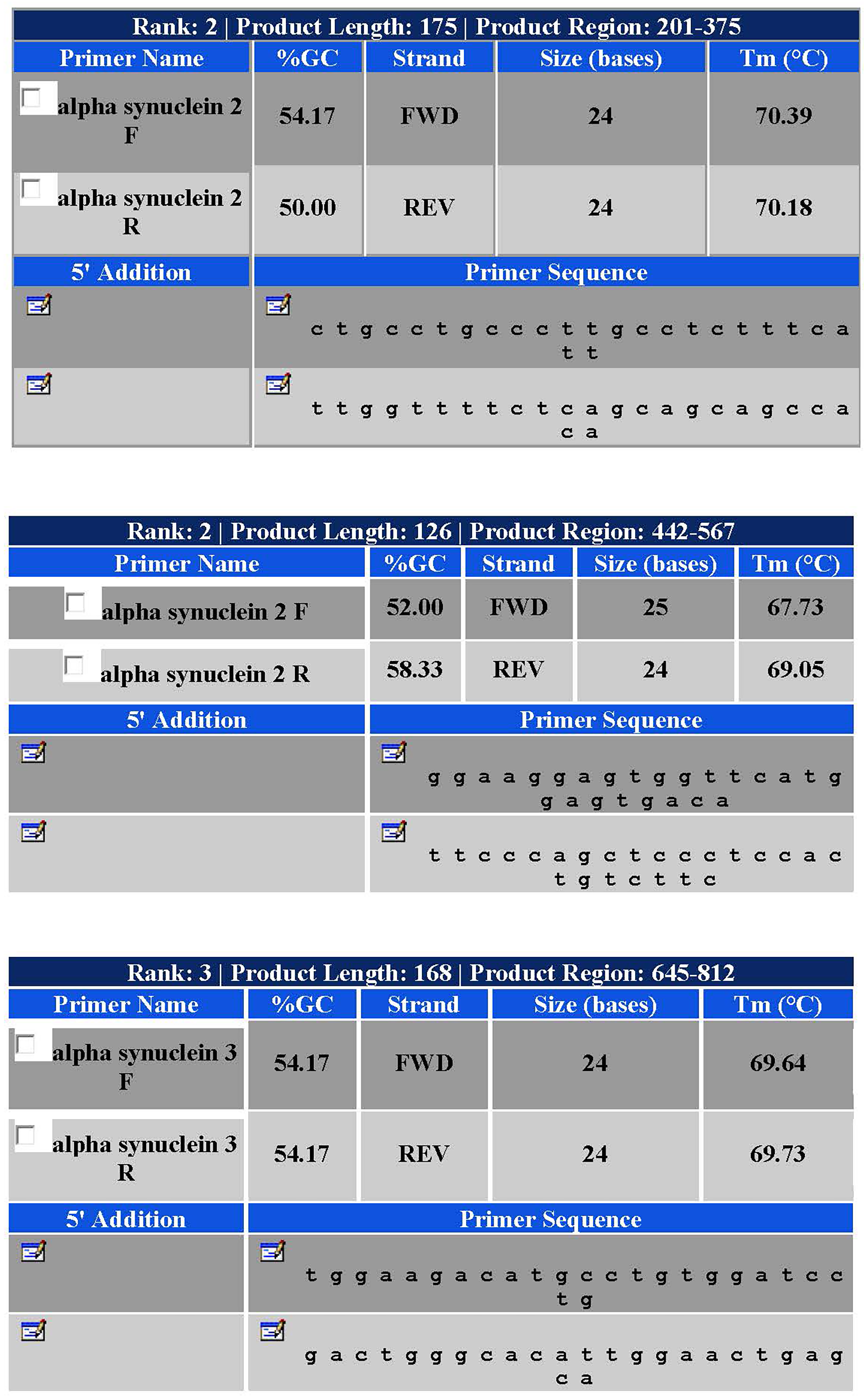

Supplement: S2 Fig — In this study, the primers sequences were designed to be useful for both cDNA and genomic DNA, with small products using cDNA and bigger products using genomic DNA. (TIF) [file pone.0222234.s002.tif]
